# Therapeutical Record

**Published:** 1854-06

**Authors:** 


THERAPEUTICAL RECORD.—*Angina Pectoris.*—M. Carriere (Bull. de Thérap., i. p. 7) has recommended the inhalation of chloroform, at the commencement of the paroxysm. Duchenne (Ibid.), in addition to this measure, has employed with advantage the "electro-cutaneous excitation" in the mammary region.

*Cataract.*—M. Lopez (Bull. Gén. de Thérap., 1854, ii. p. 89) has employed with advantage iodide of potassium taken internally, and vesication on the temples, in cataract. The treatment was persevered in for five or six months, and in three cases out of four was productive of great benefit.

*Chloroform.*—Dr. Hardy (Dublin Journal, Nov.,) relates cases to show the efficacy of chloroform vapor directed upon the part in uterine affections. The vapor is applied by means of an instrument consisting of a metallic chamber, to one end of which a pipe with a valve is attached to the gum-elastic bottle. A sponge dipped in chloroform is placed in the metallic chamber, and then by pressing on the elastic bag, the vapor is expelled through the pipe. In cases of carcinoma and simple ulceration of the os uteri, this plan appears to be very efficacious; but it is useful also in pruritus pudendi, in sore nipples, and in other painful affections of the skin. M. Nelaton (Gaz. des Hôp. and Med. Times and Gazette, March 4) relates a case in which the vapor of chloroform directed (by Hardy's apparatus) upon an abscess in the axilla, produced complete insensibility, so that the entrance of the knife was unperceived.

*Diabetes Melitus.*—Dr. Basham (Lancet, Jan.) relates two cases treated by the permanganate of potash, in doses of ten grains three times daily; the amount of urine was very slightly diminished; the sugar was augmented; the other symptous were unaffected. Dr. Basham has employed alkaline treatment with advantage; the sulphate of soda was found to be useless. In one case opium was carefully administered, to the amount of three grains daily, but no striking effect was produced.

*Fever, Continued.*—Dr. Brinton recommends at the commencement of fever, an emetic of ipecacuanha (f℥j of the tincture); afterwards a stimulant plan is adopted, consisting of the administration of *small quantities* of brandy with water, beef, tea, &c. In great abdominal pain and tympanitis, turpentine stupes and enemas are used. (For the enema the following prescription is given:—Spirits of turpentine, minims xxx; tincture of catechu, ʒij; tincture of opium, minims xv; decoction of starch, ℥ij.) The rate of mortality was 10·4 per cent.

*Fever, Intermittent.*—Dr. Harting (Schmidt's Jahrb., 1853, ix.) has employed quinoidine with alcohol and sulphuric ether in ague, and, from twelve years' experience, states that it is superior to common quinine. He considers the quinoidine to be an amorphous quinine, (an opinion which has been strongly opposed by Muller.) Dr. Castiglioni (Ibid.) has used the tannate of cinchona; it requires to be given in larger doses than quinine, but is much less expensive.

*Fever, Typhoid.*—M. Vrancken has recommended in typhoid fever, ablutions with vinegar and water. M. Van Dromme (Rev. Med. Chir., Jan. 1854,) has employed this treatment largely, and with great success. He uses one part of vinegar to three of water, applied with a sponge over the whole body night and morning.

The diet is very low; pure or slightly acidulated water is permitted to be drunk *ad libitum.* Of twenty cases treated in this manner, one died. M. Vrancken also employed the acetate of ammonia internally; but this is regarded as useless by M. Van Dromme. Chomel has also recommended the vinegar ablutions. M. Mazade (Rev. Med. Chir., Feb., p. 95, et Rev. du Midi.) has employed the sulphate of quinine, in doses of 15½ grains daily, in seventy-one cases of typhoid fever, and comes to the following conclusions:—That this remedy is eminently useful when the fever assumes a remittent form; that it is also useful, but less so, when there are less regular remissions; that it is seldom useful, and often hurtful, in typhoid fever of continued type. M. Dupré (same journal) thinks that quinine is useful in continued typhoid fever, when of very adynamic type.

*Gonorrhœa.*—Dr. Boinet (L'Un. Méd., Sept.,) speaks highly of the effect of tincture of iodine when applied to the mucous membrane of the vagina in the gonorrhœa of women; a *single* application was sometimes sufficient. At the same time a solution of equal parts of tincture of iodine and water was injected into the urethra, but was not allowed to penetrate into the bladder. Dr. Boinet has employed the local application of iodine in inflammations and ulcerations of other mucous membranes, and with great success.

*Gout.*—Dr Goolden (Med. Times and Gaz., Nov.) uses with good effect, as a local application, spirits of wine. The relief to the pain is said to be sometimes very great. In the same journal the utility of an old remedy, the carbonate of soda, as a local application in gout and rheumatism, is referred to. A drachm of the carbonate is mixed with a hot bread poultice, and applied over the joint.

*Lead Poisoning.*—Dr. Goolden (Lancet, Dec.) records a case of lead-palsy successfully treated by the iodine of potassium and by galvinism. After the commencement of the treatment the urine was examined for lead by Dr. Gladstone, who believes that lead was present, though the examination was not perfectly decisive. Dr. J. R. Nicholson (Lancet, Jan.) relates a case of lead-colic and palsy successfully treated by iodide of potassium and galvanism. The urine was examined for lead before treatment, without any being detected; during treatment, however, the lead passed off abundantly in the urine.

*Menorrhagia.*—In cases of abundant menstrual flow without physical uterine lesion, Dr. Tanner speaks highly of the effect of tincture of cinnamon, in drachm doses in cinnamon water every six hours.

*Neuralgia.*—Periodic neuralgia of the face and head have lately been common in Paris, and have occasionally withstood the action of quinine. M. Aran (Bull. Gén. de Thér., 1854, ii. p. 84) has employed in such cases the aconite in large doses. The preparation employed was the extract of the French Codex.

*Perchloride of Iron.*—As it is possible that some of our readers may be disposed to experiment with this substance, in the treatment of vascular tumors, we may remind them that the strength of the solutions used in France is regulated according to the degrees of Baumé's hydrometer. Thus, a solution is said to be 45° or 30°, and so on. Nom, a solution of 45° (Baumé), 50° Fahrenheit, is of the specific gravity 1·455; one of 36°=sp. gr. 1·26; one of 20°=sp. gr. 1·16; and one of 15°=sp. gr. 1·114. It has been shown, by M. Burin du Buisson, (Bull. Gen. de Thér. t. xlvi. p. 73), that to obtain a solution of 15°, it is not sufficient to add two parts of water to one of a solution at 45°, but it requires more than two and a half parts of water. He finds that 100 parts of the solution at 45° (sp. gr. 1·455), contain 43 parts of perchloride of iron, and 57 of water. Moreover, he states that a careful estimate of the strength of the several solutions, gives this general result—5 parts of the solution at 45° equals 10 parts at 30°, 15 parts at 20°, and 20 parts at 15°—so that any given quantity of the solution at 45° may be easily converted into either of the other strengths.

In the absence of our Surgical Report in this number, we may mention that some better success has attended the use of the perchloride of iron in the treatment of aneurism, since the introduction of the question, in November last, at the Academy of Medicine in Paris. It was then stated by M. Malgaigne, that out of 11 cases, 4 had proved fatal, and 5 or 6 had failed. A successful case, however, was then mentioned, in which M. Valette had cured a false aneurism of the brachial artery by injecting a larger quantity of solution at a higher strength than advised by M. Pravaz. He used fifteen drops at a strength of 30° Baumé, and was careful to compress the artery, for some time, above and below the point of injection. The patient has since died of some other disease. The artery was obliterated; and the sac of the aneurism filled with firm clot containing particles of chloride of iron.

Experiments in the zygomatic, external plantar, and carotid arteries of the horse, have been made by MM. Debout and Leblanc, with success, and without any bad consequences. They employed at last, a solution at 15°, and insist on the necessity for temporary compression of the vessel. The perchloride solution has also been injected into varicose veins by M. Follin, and into a *venous* tumour (query erectile) by M. Giraldis, both with success. It has also been employed, by M. Thierry, externally upon varicose veins, and varicose ulcers, as well as in a case of lupus of the face, and in another of nævus. In all cases the results were good. Where the epidermis is entire, a blister is first applied. Finally, the solution has been applied to internal hæmorrhoids; and, with complete success, by M. Miergues, in a case of fistula in and communicating with the bowels. We shall await further trials with this agent, and consider the subject again hereafter.

*Phosphate of Lime.*—M. Mourié's (Compte-rendu de l'Acad., Jan.) states that in the French towns the food is deficient in phosphate of lime. He affirms this after examination of the food and of the urine of women; the ingestion and egestion of this substance are diminished by a fourth. He also affirms that in the milk of nursing women, fed on this diet, there is a great deficiency of phosphate of lime, and consequently the children born in the great cities are insufficiently nourished. He recommends, therefore, the administration of phosphate of lime, and in nursing women he has given it with benefit to the amount of ʒiii per diem. [M. Mourié's conclusions appear to be based on an insufficient number of observations, but they accord with the researches of Beneke, (Zur Phys. und Path. des Phosphorsauren Kalks, Gött., 1850), who has also rendered extremely probable the relation of phosphate of lime to the formation of cells.]

*Pityriasis Capitis.*—Dr. May, (Lancet, Sept.) recommends a lotion of biborate of soda (℥ss.), camphor (ʒij.), and water (℥xxxij.). Twice a week the scalp is gently wiped with a soft flannel saturated with the solution. M. Duplex (Lancet, Oct.) advises the nitrate of mercury ointment, mixed with a little olive oil, to make it more manageable. Mr. O'Connor (Lancet, Oct.), who has tried the biborate of soda without effect, recommends washing the head in cold water, and the administration, by the mouth, of the sesquicarbonate of soda in some bitter infusion. Mr. Wingar (Lancet, Oct.) speaks highly of the following lotion:—fresh sulphuret of potassium (ʒj), water (℥iij); to be used daily.

*Phthisis.*—M. Piorry (Bull. Gén. ne Thér., 1854, ii. p. 86) has employed, in 31 cases of phthisis, inhalations of iodine. About thirty grains of iodine are placed in a large jar with a wide opening, and the patient places the mouth over the opening, and inhales deeply one or two hundred times in the twenty-four hours, or even more often than this. At other times a larger quantity of iodine is exposed in the room, so as to impregnate the atmosphere. M. Piorry declares that not only are the cough, expectoration, &c., improved, but that the signs on percussion (of which method M. Piorry is, as is well known, an ardent votary) also improve. The editor of the "Bull. de Thér." expresses doubts of the validity of these conclusions.

*Pneumonia.*—In old decrepit persons, in tuberculous subjects, in those who are suffering at the time from diarrhœa, Dr. Fielding (Schmidt's Jahrb., 1853–4, ix.) recommends acetate of lead (three to six grains) with digitalis and opium. Heusinger (Deutsche Klinik, 1853, xxiv.) has employed digitalis at the very commencement, or after one or two doses of tartar emetic. In twenty-four to thirty hours the physiological effects of the digitalis appear—viz: weakness, purging, vomiting cold moist skin, slowness and intermission of the pulse, &c. When thess symptoms appear, the pneumonic process is arrested, and in a few hours resolution commences. The digitalis is given as infusion, in moderate doses, every hour or every two hours. M. Aran (L'Union Médicale, September, et Bull. de Thérap.) has employed *veratrine* in inflammatory affections, in typhoid fever, in acute rheumatism, and especially in pneumonia. M. Aran had employed veratrine in rheumatiem after the manner of Piedagnel and Trousseau (vide de Thérap. Record, July, 1853, p. 282), and being struck by the enormous decrease in the rapidity of the pulse (from July 112 to 64), determined to employ the medicine in pheumonia. Six cases are related. One died; the rest were rapidly cured. After 5, 10, or 15 milligrammes (one-fourteenth, one seventh, and two-sevenths of a grain) of veratrine given in divided doses, the general symptoms began to abate. The effect on the pulse was extremely marked; it fell in an extraordinary way. The number of respirations diminished; the action on the nervous system was marked by extreme debility, prostration, pallor of the face, &c. M. Aran, however, does not pronounce exclusively in favor of this treatment but would employ it in obstinate cases. He gives five milligrammes (one-quarter of a grain) every four or six hours the first day, and in gradually lessening quantity the three succeeding days.

*Priapism.*—Dr. Debont (Gaz. des Hôp., 1853, p. 61) calls attention to the efficacy of the tincture of hops, in priapism. The effect seems heightened by combining sugar with it.

*Ptyalism.*—Dr. Erpenbeck (Schmidt's Jahrb., 1853, p. ix.) relates a case of severe mercurial salivation arrested by the internal use of belladonna. The salivation returned when the remedy was discontinued, and was again checked by it.

*Sciatica.*—Mr. Hancock believes that most cases of sciatica are caused by pressure on the nerve within the pelvis, either by accumulation in cæcum and colon, or by tumors. He recommends croton oil internally to remove fæcal accumulations, in doses of half a drop combined with blue pill. Quinine is to be given after the croton oil has fully operated.

*Spermatorrhœ.*—Dr. Laroche (Bull. Gén. de Thér., 1854, II. p. 76) records a case of spermatorrhœa treated with success by digitalis, as recommended by Corvisart. The dose is one or two grains of the powder of the plant, gradually elevated to eight grains.

*Spleen, Tumor of.*—In a case of splenic tumor, Dr. Gurtrac (L'Un. Méd., 1853, No. 69) has employed the sulphate of manganese with good effect, in doses of one to one and a half grains twice a day, in the form of pill.

*Strabismus.*—Du Bois Reymond (Muller's Archiv. and Bull. Gén. de Thér. 1854, II. p. 95) recommends the *stereoscope* for the cure of squinting. The principle is the same as in the case of the prismatic spectacles, advised by Mr. Spencer Wells.

*Syphilis.*—Dr. Heyfelder (Medical Times and Gazette, Oct.) has employed the bichromate of potash in secondary syphilis, with good effect. The dose is two-thirds of a grain daily for a few days and then the quantity is gradually increased to three and four grains, until altogether about 130 grains have been taken.

*Tape-worm.*—Dr. Mackinnon (Indian Annals of Med. Science, No. 1., p. 284) recommends a remedy called in Northern India the "Kameyla," and used as a vermifuge for dogs. He has given it in 16 cases, and finds it more useful than turpentine or kousso. The dose is two or three drachms, which purges rather copiously. The "Kameyla" is a powder attached to the fruit-capsule of an Euphorbiaceous plant, the "Rothera Tinctoria;" it is brushed off and collected when the fruit is dry; its price is moderate.

*Ulcers.*—In callous ulcers, Mr. Hainworth has successfully adopted the plan of paring away the ring of hard condensed cuticle which surrounds the old ulcer. The operation is painless, for the cuticle is sliced off without the cutis being wounded.

*Vomiting.*—MM. Clertan and Galante (Bull. Gén. de Thér., ii. p. 91) have employed, in cases of obstinate chronic vomiting which had resisted all other treatment, capsules containing sulphuric ether. In a case of apparently hysterical vomiting, which is related as evidence, ether taken in the ordinary way did no good, but the disengagement of the ether in the stomach was at once successful.—*Medico-Chirurgival Review.*

